# Transcriptional consequences of trisomy 21 on neural induction

**DOI:** 10.3389/fncel.2024.1341141

**Published:** 2024-01-30

**Authors:** José L. Martinez, Jennifer G. Piciw, Madeline Crockett, Isabella A. Sorci, Nikunj Makwana, Carissa L. Sirois, Yathindar Giffin-Rao, Anita Bhattacharyya

**Affiliations:** ^1^Waisman Center, University of Wisconsin-Madison, Madison, WI, United States; ^2^Cellular and Molecular Biology Graduate Program, University of Wisconsin-Madison, Madison, WI, United States; ^3^Medical Scientist Training Program, School of Medicine and Public Health, University of Wisconsin-Madison, Madison, WI, United States; ^4^Department of Cell and Regenerative Biology, School of Medicine and Public Health, University of Wisconsin-Madison, Madison, WI, United States

**Keywords:** Down syndrome, iPSC, neurodevelopment, RNAseq, neural induction

## Abstract

**Introduction:**

Down syndrome, caused by trisomy 21, is a complex developmental disorder associated with intellectual disability and reduced growth of multiple organs. Structural pathologies are present at birth, reflecting embryonic origins. A fundamental unanswered question is how an extra copy of human chromosome 21 contributes to organ-specific pathologies that characterize individuals with Down syndrome, and, relevant to the hallmark intellectual disability in Down syndrome, how trisomy 21 affects neural development. We tested the hypothesis that trisomy 21 exerts effects on human neural development as early as neural induction.

**Methods:**

Bulk RNA sequencing was performed on isogenic trisomy 21 and euploid human induced pluripotent stem cells (iPSCs) at successive stages of neural induction: embryoid bodies at Day 6, early neuroectoderm at Day 10, and differentiated neuroectoderm at Day 17.

**Results:**

Gene expression analysis revealed over 1,300 differentially expressed genes in trisomy 21 cells along the differentiation pathway compared to euploid controls. Less than 5% of the gene expression changes included upregulated chromosome 21 encoded genes at every timepoint. Genes involved in specific growth factor signaling pathways (WNT and Notch), metabolism (including oxidative stress), and extracellular matrix were altered in trisomy 21 cells. Further analysis uncovered heterochronic expression of genes.

**Conclusion:**

Trisomy 21 impacts discrete developmental pathways at the earliest stages of neural development. The results suggest that metabolic dysfunction arises early in embryogenesis in trisomy 21 and may affect development and function more broadly.

## 1 Introduction

The development of the nervous system begins with neural induction whereby pluripotent cells become restricted to a neuroectodermal fate. Neuroectoderm undergoes further development to generate neuroepithelial cells, or neural stem cells, that give rise to neural progenitor cells that will ultimately differentiate into neural cell types (neurons, astrocytes, oligodendrocytes). The ability to assess the early events of human neural induction is limited by the lack of accessibility to human embryonic tissue. *In vitro* culture of human pluripotent stem cells (PSCs) provides a means to analyze these early timepoints as neuroepithelial differentiation from human PSCs resembles *in vivo* neuroectoderm induction in its temporal course, morphogenesis, and biochemical changes (Pankratz et al., 2007; Lavaute et al., 2009; Kamiya et al., 2011; Huang et al., 2016).

Alterations in any of the processes of neural development can result in neurodevelopmental disorders that are characterized by intellectual disability. In fact, genetic studies indicate that autism-related genes overlap with genes critical for all stages of neural development, including early neural induction (Casanova and Casanova, 2014), reflecting the importance of fidelity in these processes. The most common genetic cause of intellectual disability is Down syndrome (DS) caused by trisomy 21 (T21). Reduced neurogenesis and cortical size have been established in mid-late gestation fetuses and neonates with T21, indicating changes to prenatal neural development (Ross et al., 1984; Wisniewski et al., 1984; Schmidt-Sidor et al., 1990; Golden and Hyman, 1994; Guihard-Costa et al., 2006; Guidi et al., 2008; Guidi et al., 2011; Guidi et al., 2018; Stagni et al., 2019; Baburamani et al., 2020; Patkee et al., 2020; Tarui et al., 2020; McCann et al., 2021). Yet, these results represent endpoints of neural development, and there is little to no information about the impact of T21 on the earliest stages of nervous system formation.

Induced PSCs (iPSCs) generated from individuals with DS enable modeling of neural development in a disorder whose genetic basis is not easily reproduced in animal models (Gardiner and Davisson, 2000; Antonarakis, 2001; Sturgeon and Gardiner, 2011; Hibaoui et al., 2014). Here, we use this powerful cellular paradigm to address a key gap in understanding the impact of T21 on early neural development. Using T21 and isogenic euploid control iPSCs, we interrogated the molecular impact of T21 on neural induction using bulk RNA sequencing. Our results reveal that T21 dysregulates WNT signaling and increases inflammatory response and oxidative stress, highlighting the impact of T21 on initial stages of neural development.

## 2 Materials and methods

### 2.1 Cell culture and differentiation

One pair of trisomy 21 and euploid control iPSCs were generated from fibroblasts from a 24-year-old female mosaic for trisomy 21 (Weick et al., 2016; Giffin-Rao et al., 2022). iPSCs undergo regular karyotyping and mycoplasma testing to ensure quality (WiCell Institute, Madison WI). iPSCs were maintained on mouse embryonic fibroblasts (MEFs) feeder plates and differentiated to definitive neuroectoderm following previously published studies (Zhang et al., 2001; Pankratz et al., 2007; Chambers et al., 2009). Briefly, iPSC differentiation was initiated by detaching iPSC colonies with dispase to form aggregates [embryoid bodies (EBs)] at day 0. EBs were fed daily with hEB media (DMEM/F12, 20% knockout replacement serum, non-essential amino acids, L-glutamine, and β-mercaptoethanol) with dual SMAD inhibitors (SB-431542 and LDN-193189 2HCl) for 4 days. From days 4 to 7, EBs were maintained in neural induction media (NIM) (DMEM/F12, N2 supplement, non-essential amino acids, and heparin) and fed every other day for 3 days. At day 7, EBs were allowed to attach to plates using NIM and 5% FBS for approximately 6–8 h before the media was changed to NIM. Plated EBs were fed every 2–3 days with NIM until neural rosettes, indicative of neural stem cells, emerged (∼Day 14) when they are detached and grown in suspension as neurospheres (NS) and maintained in NIM media. Three technical replicates (batches of differentiation) were carried out for each experiment.

For additional information on cell culture and differentiation reagents, please refer to Supplementary Table 1.

### 2.2 Total RNA isolation and bulk RNA sequencing

Total RNA was isolated from all time points using Direct-zol RNA Micro Prep Kit (Zymo Research) according to manufacturer’s directions. Samples were eluted with nuclease free water and validated for quality based on 260:280 values and concentration. RNA sample quality, preparation of a 1 × 100 bp stranded mRNA poly-A tail enriched unpaired end library preparation and RNA sequencing was performed by the Biotechnology Center at the University of Wisconsin—Madison on the Illumina HiSeq 4000 platform. Refer to Supplementary Tables 1, 2 for additional information on RNA isolation.

### 2.3 Data analysis

Bulk RNA-seq resulting reads were aligned to the human genome (GRCh37/hg19) and gene counts extracted using the Spliced Transcripts Alignment to a Reference (STAR v2.7.11a) package (Dobin et al., 2013). Count files were transferred to RStudio (RStudio v3.5.3)^1^ and merged into a single table per experimental timepoint. Zero count genes and the bottom 10% of genes with counts were removed. Counts were normalized by size factors and differentially expressed genes (DEGs) analysis was done using DESeq2 v1.22.2 (Love et al., 2014). DEGs were obtained by comparing the results of the isogenic control cells against the results of the trisomy 21 cells. Principal Component Analysis was performed on normalized counts using plotPCA in ggplot2 v3.3.5 package in RStudio. Thresholding for DEGs was established with an adjusted *p*-value < 0.05 and Log2FoldChange > 1.0. The RStudio package pheatmap v1.0.12 was used to plot the heatmaps and hierarchical clustering was done using the distance matrix with Pearson’s correlation. Volcano plots were generated with the Enhancedvolcano v1.13.2 package in RStudio to visualize significant DEGs. The top ten DEGs based on adjusted *p*-value were highlighted in the plots. All Venn diagrams were generated with the ggvenn v0.1.10 package in RStudio. Gene expression profiles of our identified clusters were generated using the ggplot2 v3.3.5 package in RStudio implementing a linear model method.

### 2.4 GO-term and pathway analysis

Differentially expressed genes were filtered by adjusted *p*-value < 0.05 and a log2FoldChange > 1 using RStudio. Further filtration was done by removing genes that did not have a count greater than zero throughout the entirety of our differentiation timepoints. The resulting 96 genes were further divided into 4 gene lists that represent the identified expression profile clusters in our heatmap hierarchical clustering analysis. Enrichr^2^ was used to perform a comprehensive GO-term, KEGG, and Pathway Analysis (Chen et al., 2013; Kuleshov et al., 2016; Xie et al., 2021). Separate Enrichr analyses were performed for each of the identified cluster gene list. For additional information on genes in identified clusters refer to Supplementary Table 3, and for Enrichr analysis refer to Supplementary Tables 4–7.

### 2.5 qPCR

Total RNA was isolated from three replicates of differentiation for all time points using Direct-zol RNA Micro Prep Kit (Zymo Research) according to manufacturer’s directions. A total of 500 ng of total RNA was used to make cDNA using qScript cDNA SuperMix kit (Quantabio). qPCR was performed in triplicate on 3 batches of differentiation (*N* = 3, *n* = 3) using iTaq Universal SYBR Green Supermix (Bio-Rad) on CFX Connect Real-Time System (Bio-Rad). Data are presented as ddCt values. Error bars indicate ddCt values ± 1 SD. Statistical significance was determined by two-way ANOVA on ddCt values. Primer sequences for all amplicons can be found in Supplementary Table 3.

## 3 Results

### 3.1 Identification of transcriptional differences between isogenic trisomy 21 and control iPSCs during neural induction

**FIGURE 1:**
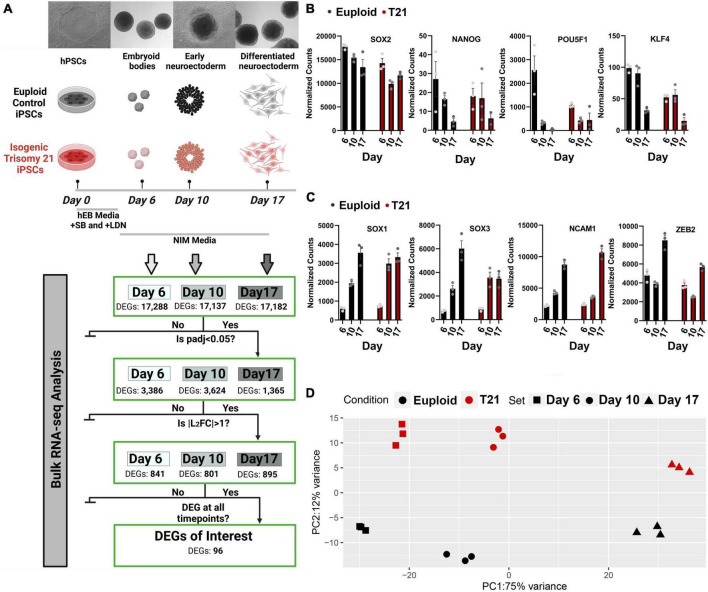
Transcriptional differences between isogenic trisomy 21 and euploid iPSCs during neural induction. **(A)** Experimental design of control and isogenic T21 human iPSCs (WC-24-02) differentiated to dorsal forebrain neural progenitor cells and analyzed by RNA sequencing at day 6, day 10, and day 17. **(B)** Expression of stem cell marker genes SOX2, NANOG, POU5F1, and KLF4 assessed by normalized bulk RNAseq counts at each time point. **(C)** Expression of neuroectoderm genes SOX1, SOX3, NCAM1, and ZEB2 in samples assessed by normalized bulk RNAseq counts at each time point. **(D)** Principal component analysis of trisomy 21 (red) and isogenic control (black) at all-time points. Created with Biorender.com.

To identify transcriptional consequences of T21 during neural induction, a pair of control and isogenic T21 human iPSCs were differentiated to dorsal forebrain neural progenitor cells (Figure 1A) and analyzed by bulk RNA sequencing. To confirm neural differentiation, the expression of stem cell marker genes SOX2, NANOG, POU5F1, and KLF4 was assessed. As expected, stem cell marker gene expression was downregulated in both euploid and T21 cells as they differentiated (Figure 1B). Concurrently, the expression of neuroectoderm genes SOX1, SOX3, NCAM1, and ZEB2 was upregulated in both euploid and T21 cells as they differentiated (Figure 1C). The downregulation of stem cell marker genes and upregulation of neuroectoderm genes confirmed the conversion of iPSCs to neurepithelium in culture (Huang et al., 2016) Created with Biorender.com.

**FIGURE 2:**
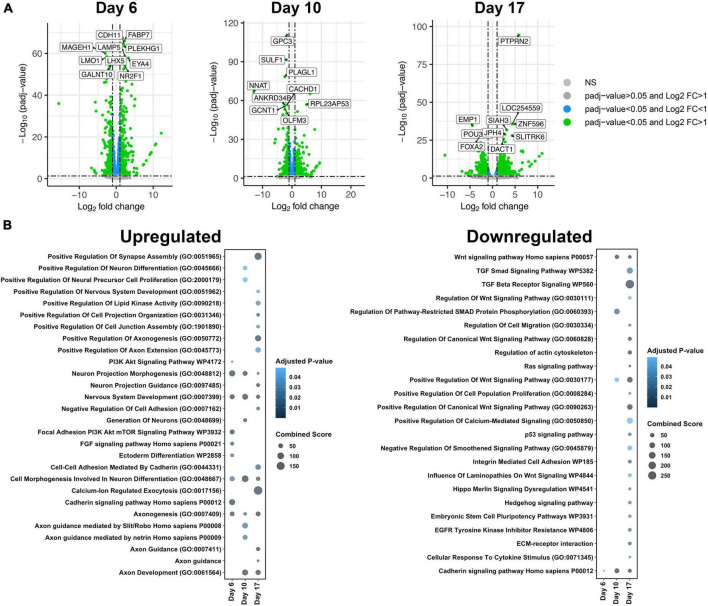
Gene ontology (GO) analysis of differentially expressed genes (DEGs) between isogenic trisomy 21 and euploid cells at each time point. **(A)** Volcano plots showing DEGs with padj < 0.05 and L2FC > 1 in green, padj < 0.05 in blue, and padj > 0.05 in gray. The top 10 DEGs based on padj are labeled at each timepoint. **(B)** Bubble plot highlighting unique GO Terms of upregulated genes and downregulated genes per timepoint (day 6, day 10, or day 17).

Bulk RNA sequencing of samples was performed at three timepoints: day 6 embryoid bodies, day 10 early neuroectoderm and day 17 differentiated neuroectoderm (Figure 1A). Principal component analysis (PCA) of gene counts revealed that the samples segregate by stage along neural induction (75% variance) and cluster by disorder (T21 vs. control) at each timepoint (12% variance) (Figure 1D). Heatmap hierarchical clustering of samples by transcriptomic data shows that samples segregated into control and T21 at each individual timepoint (Supplementary Figures 1A–C). These results indicate that gene expression changes driven by T21 can be detected during the process of neural induction. Differentially expressed genes (DEGs) in T21 vs. control were identified at each timepoint (Figure 1A). DEG analysis and hierarchical clustering reveal significant differences in expression profiles between T21 and control at each stage: Day 6/EBs (17,288 genes), Day 10/early neuroectoderm (17,137 genes) and Day 17/differentiated neuroectoderm (17,182 genes; Figure 2A and Supplementary Figures 1A–C). Using stringent cutoffs of adjusted *p*-value (padj < 0.05) and log2FoldChange (| L2FC| > 1), we identified 841 DEGs at day 6, 801 DEGs at day 10, and 895 DEGs at day 17 (Figures 1A,2A). A total of 96 DEGs are consistently dysregulated throughout the time course (Figure 1A) suggesting that they play a continuous role in neural induction in the context of T21.

### 3.2 Timepoint specific DEGs suggests impacts on differentiation and neurogenesis

In addition to DEGs that were constantly mis-expressed in T21 cells across differentiation, unique DEGs were identified at each time point (Figure 2A, Supplementary Figure 1 and Supplementary Tables 4–6). To gain insight into the impact of the DEGs at each time point, enrichment analysis was performed (Chen et al., 2013; Kuleshov et al., 2016; Xie et al., 2021; Figure 2B). Molecular pathways and biological processes related to neural differentiation, extracellular matrix, and Wnt signaling were the primary pathways altered in T21 cells (Figure 2B). DEGs were separated into those that were upregulated, generally suggesting activation or prominence of pathways, and those that were downregulated, suggesting suppression or inhibition of pathways. Upregulated pathways were evident as early as day 6, while down regulated pathways generally emerged at day 10 and Day 17, suggesting that activation of specific pathways occurs as early as day 6 in T21.

Upregulated pathways at all-time points were associated with nervous system development and cell morphogenesis involved in neural differentiation, which was expected as our experimental paradigm is neural development. However, the upregulation of these pathways in T21 suggests that T21 progenitor cells are activating differentiation programs precociously (Figure 2B and Supplementary Table 4). The earliest pathways that emerge from this analysis are the phosphatidylinositol 3-kinases protein kinase B (PI3K Akt) and FGF signaling pathways that together are driven largely by expression of FGF and other growth factor ligands and receptors and cyclins. Altered PI3K Akt signaling may also affect other functions in T21 neural progenitors as it is a highly conserved process controlling cell metabolism, growth, proliferation, and survival and is activated by many signals in the nervous system (Hemmings and Restuccia, 2012; Sánchez-Alegría et al., 2018). These pathways are critical in regulating neurogenesis and differentiation and so the results support altered neurogenesis.

In contrast, downregulated pathways at all-time points were associated with growth factor signaling, primarily Wnt and TGF beta. Only the cadherin pathway is down regulated at Day 6, while Wnt is downregulated at day 10 and others (TGF beta and HH) at Day 17 (Figure 2B and Supplementary Tables 5, 6). Thus, day 10 may be a critical time point in the dysregulation of Wnt signaling in neural induction in T21. These growth factors act as mitogens and morphogens during neural induction and so the downregulation may support decreased proliferation of T21 cells or their premature exit from the cell cycle.

### 3.3 Few differentially expressed genes are encoded by Hsa21

**FIGURE 3:**
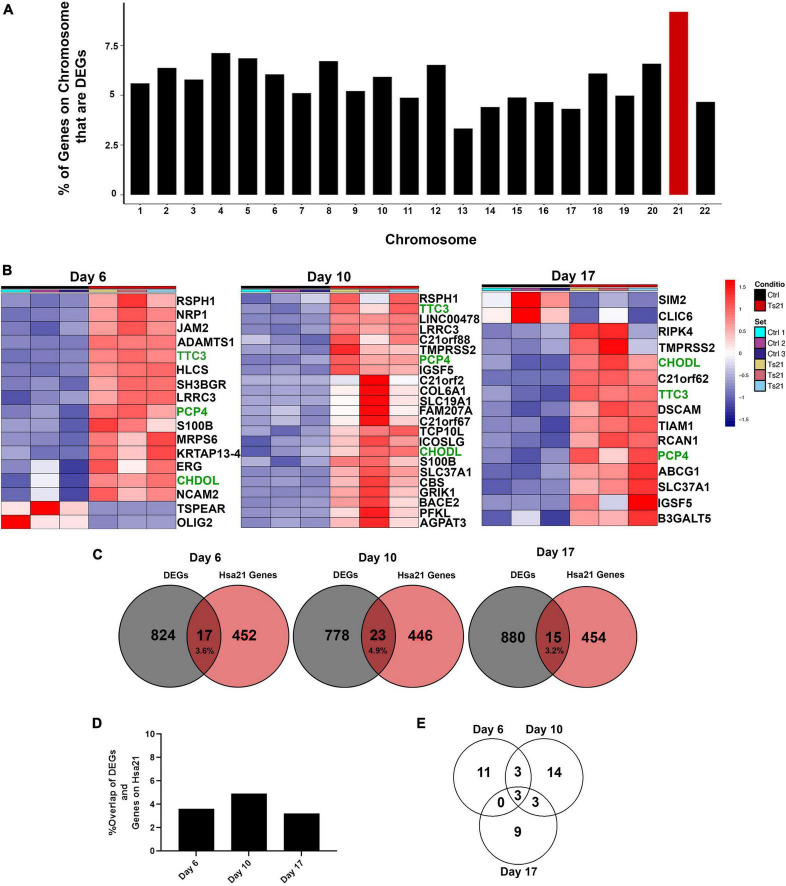
Differential gene expression of HSA21 encoded genes. **(A)** Graph representing the percent of genes per chromosome that are significant DEGs. **(B)** Heatmap of differentially expressed chromosome 21 genes using DEGs with a padj < 0.05 and L2FC > 1. Time course replicates are labeled with light gray for day 6, medium gray day 10, and dark gray day 17. Isogenic control is represented by black and T21 by red. Upregulated genes are displayed in red while downregulated genes are expressed in blue, genes that are present at all timepoints are highlighted in green text. **(C)** Venn diagram showing the number of HSA21 genes overlapping throughout the timepoints. Number of unique Hsa21 genes in red and number of non-overlapping DEGs in gray, overlapping area shows percentage overlap. **(D)** Graph showing percent overlap at each timepoint. **(E)** Venn diagram showing the number of Hsa21 genes overlapping with DEGs that have a padj < 0.05 and L2FC > 1.

Genes encoded by human chromosome 21 (Hsa21) are expected to have increased expression due to gene dosage. To assess the relative contribution of Hsa21 genes, we sorted the results of our differential gene expression analysis by chromosome. Genes encoded in all chromosomes were differentially expressed (Figure 3A). As expected, Hsa21 has the highest percentage of DEGs based on the proportion of genes on each individual chromosome (Figure 3A). Only 17 Hsa21 genes at day 6, 23 Hsa21 genes at day 10, and 15 Hsa21 genes at day 17 meet our criteria (Figures 3B, C). Although these Hsa21 encoded DEGs are generally overexpressed, TSPEAR and OLIG2 at day 6, and SIM2 and CLIC6 at day 10 were down regulated (green in Figure 3B). Hsa21 DEGs represented 3.6% of all DEGs at day 6, 4.9% of all DEGs at day 10, and 3.2% of all DEGs at day 17 (Figures 3C, D). Only 3 Hsa21 genes (PCP4, TTC3, and CHODL) were expressed throughout the differentiation (Figure 3E). The low percentage of dysregulated Hsa21 genes indicates that T21 has broad transcriptional consequences across the genome early in neural induction.

### 3.4 Genes with constant expression and dynamic expression patterns reveal four unique clusters

**FIGURE 4:**
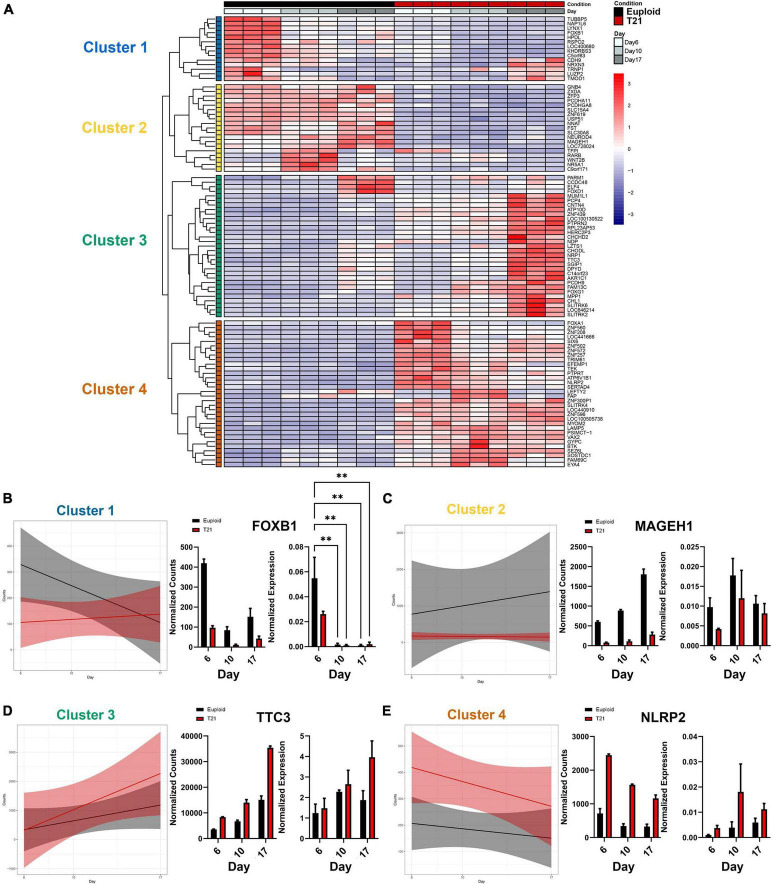
Transcriptional changes in T21 reveal four major expression patterns. **(A)** Heatmap of differentially expressed genes using DEGs with a padj < 0.05 and L2FC > 1. Hierarchical clustering reveals four clusters. Time course replicates are labeled with light gray for day 6, medium gray day 10, and dark gray day 17. Isogenic control is represented by black and T21 by red. Upregulated genes are displayed in red while downregulated genes are expressed in blue. **(B–E)** Plots showing linear model of the average expression trends of DEGs in each cluster over the time course of differentiation to neuroectoderm. Graphs show gene expression validation for representative genes over the time course for each cluster with normalized bulk RNAseq counts on the left and normalized expression of quantitative PCR (*N* = 3 batches, *n* = 3 technical replicates). Error bars indicate ddCt values ± 1 SD. Statistical difference was determined by two-way ANOVA on ddCt values. **p < 0.01.

To explore the transcriptional impact of T21 throughout neural induction, we performed unbiased hierarchical clustering of our 96 DEGs to identify patterns of altered regulation. Results reveal four clusters with unique and dynamic expression profiles, identified through linear model analysis (Figure 4A and Supplementary Table 7). Cluster 1 genes are highly expressed at day 6 and their expression decreases over time in euploid cells, while the genes have low expression in T21 cells (Figures 4A, B). Cluster 2 identified genes expressed in euploid cells and not expressed in T21 cells throughout differentiation (Figures 4A, C). Cluster 3 revealed a heterochronic pattern of gene expression in which the genes are expressed earlier in T21 cells compared to euploid cells (Figures 4A, D). The last cluster, Cluster 4, represents genes that are upregulated in T21 cells compared to euploid cells at all differentiation time points (Figures 4A, E). The expression pattern of one gene from each cluster was validated by qPCR (Figures 4B–E). These distinctive expression patterns suggest that genes in each cluster have a unique impact driven by T21 during neural induction.

### 3.5 Cluster specific gene ontology (GO) and pathway analysis

**FIGURE 5:**
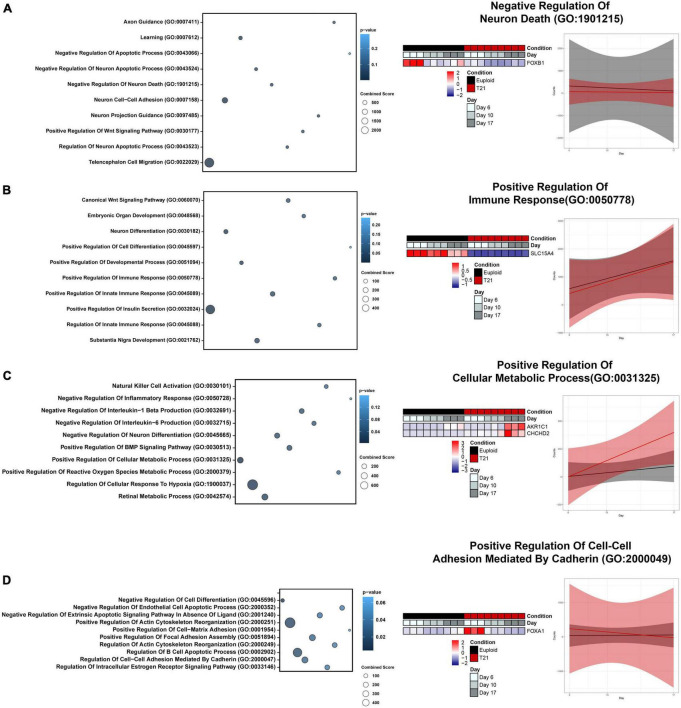
Gene ontology (GO) Analysis of four major expression patterns. **(A)** Bubble plot highlighting unique GO Terms to Cluster 1, relating to brain development and regulation of cell death. Heatmap and gene expression trend linear model of representative GO Term “Negative Regulation Of Neuron Death (GO:1901215).” **(B)** Bubble plot highlighting unique GO Terms to Cluster 2, relating to immune response. Heatmap and gene expression trend linear model of representative GO Term “Positive Regulation Of Immune Response (GO:0050778).” **(C)** Bubble plot highlighting unique GO Terms to Cluster 3, relating to inflammation response and metabolism. Heatmap and gene expression trend linear model of representative GO Term “Positive Regulation Of Cellular Metabolic Process (GO:0031325).” **(D)** Bubble plot highlighting unique GO Terms to Cluster 4, relating to neurotrophic signaling and extracellular matrix. Heatmap and gene expression trend linear model of representative GO Term “Positive Regulation Of Cell-Cell Adhesion Mediated By Cadherin (GO:2000049).”

To gain insight into the impact of the gene expression patterns revealed by hierarchical clustering, unbiased functional enrichment analysis was performed using Enrichr (Chen et al., 2013; Kuleshov et al., 2016; Xie et al., 2021) to identify molecular pathways and biological processes that are represented by each cluster. Enrichment analysis identified key biological processes including regulation of cell death, immune response, inflammation response, metabolism, and extracellular matrix that are impacted by T21 during neural differentiation (Figure 5). Cluster 1 contained genes in pathways related to brain development and regulation of cell death, which was anticipated given that our experimental paradigm is neural development (Figure 5A and Supplementary Table 8). Decreased expression of genes regulating these pathways in T21 foreshadow neurodevelopmental defects in DS and suggest that there may be increased neuronal death throughout neural development in DS. Cluster 2 was enriched for terms related to innate immune response (Figure 5B and Supplementary Table 9). Interactions between the immune system and neuronal cells influence processes critical for neural development, including synapse remodeling. The onset and impact of decreased gene expression in these pathways on neurodevelopment in T21 remain unclear as our culture paradigm does not include interactions with immune cells. Nonetheless, the results suggest that T21 neural progenitor cells have intrinsic dysregulation that may impact response to inflammation early in neural induction. Cluster 3 shows enrichment of genes in inflammation response and metabolism pathways (Figure 5C and Supplementary Table 10), indicating T21 causes an early dysregulation of metabolic processes. Cluster 4 highlights a dysregulation in the extracellular matrix (ECM) early in neural induction (Figure 5D and Supplementary Table 11). Disruptions in ECM can affect many aspects of neural development (Long and Huttner, 2019), and these results may predict multiple processes that are defective in DS and other neural development disorders (Long and Huttner, 2021). Taken together, these results indicate that distinct gene expression patterns in T21 cells regulate separate functional pathways, many of which have been previously implicated in DS.

### 3.6 Decreased Wnt signaling in T21 neural induction

**FIGURE 6:**
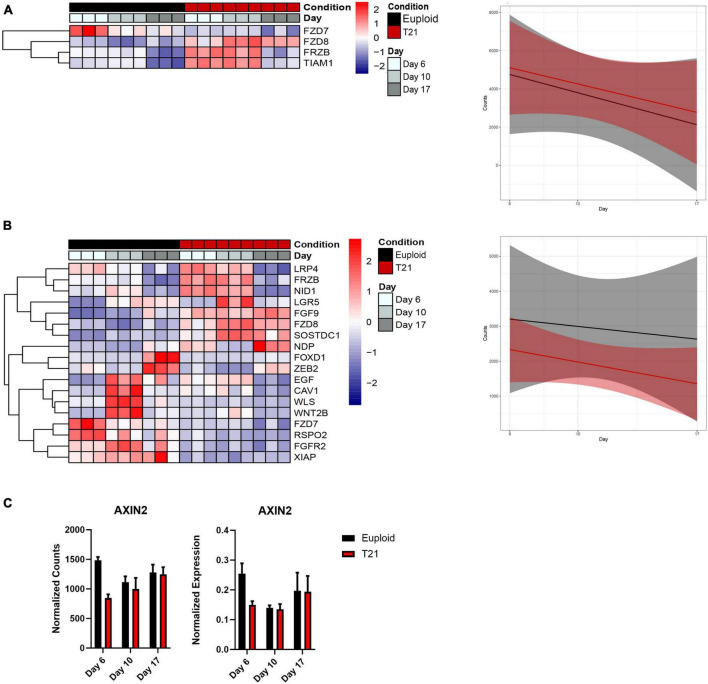
Wnt signaling in T21 neural induction. **(A)** Heatmap and plot showing linear model of the average expression trends of differentially expressed in non-canonical Wnt signaling pathway genes that have an adjusted *p*-value < 0.05. Time course replicates are labeled with light gray for day 6, medium gray day 10, and dark gray day 17. Isogenic control is indicated by black and T21 by red. Upregulated genes are displayed in red while downregulated genes are expressed in blue. **(B)** Heatmap and plot showing linear model of the average expression trends of differentially expressed canonical Wnt signaling pathway genes that have an adjusted *p*-value < 0.05. Time course replicates are labeled with light gray for day 6, medium gray day 10, and dark gray day 17. Isogenic control is represented by black and T21 by red. Upregulated genes are displayed in red while downregulated genes are displayed in blue. **(C)** AXIN2 gene expression shown by normalized RNAseq counts and qPCR.

The impact of T21 on Wnt signaling emerges from analysis of clusters 1, 2, and 3, so we specifically explored the dysregulation of Wnt signaling pathways in our data. Using a lower threshold of an adjusted *p*-value < 0.05 revealed dysregulation in both canonical and non-canonical WNT signaling pathways in T21 (Figure 6). Of note, we did not externally modulate WNT signaling with XAV939, an antagonist of the canonical pathway known to promote neural specification.

Analysis of the non-canonical Wnt pathway revealed altered expression of only four genes. The trajectories of these four genes indicated an increase in expression in T21 compared to euploid during the course of neural induction (Figure 6A). Three of these genes are Wnt receptors that also part of the canonical Wnt pathway (FZD7, FZD8, FRZB) (Figure 6A). It is therefore unclear whether the non-canonical Wnt pathway is specifically affected in T21. The fourth gene is TIAM1, a RAC1-specific guanine nucleotide exchange factor encoded on Hsa21. TIAM1 regulates RAC1 signaling pathways that affect cell shape, migration, adhesion, growth, survival, and polarity, as well as influencing actin cytoskeletal formation, endocytosis, and membrane trafficking. The early overexpression of this gene may suggest that it affects functions in addition to the non-canonical Wnt pathway.

Analysis of the canonical (β-catenin) Wnt signaling pathway identified 18 genes as differentially expressed in T21 at all-time points (Figure 6B), with all 18 genes showing decreased expression throughout differentiation in both T21 and euploid, but less expression in T21 compared to euploid. To validate that the Wnt signaling pathway was decreased in our T21 cells, we assessed the expression of AXIN2, a Wnt target gene. Results show that AXIN2 expression is decreased at day 6 in T21 cells (Figure 6C). These results agree with the results from analyses in Figures 2 and 5 that suggest reduced Wnt signaling in T21.

## 4 Discussion

### 4.1 Transcriptional dysregulation by T21 during neural induction

We leveraged *in vitro* iPSCs models of Down syndrome to assess the transcriptional impact of T21 on the process of neural induction, building on seminal work modeling *in vivo* human neural development with hESCs (Zhang et al., 2001; Pankratz et al., 2007; Lavaute et al., 2009). These stages are inaccessible in human embryos, although the emergence of PSC-derived embryo models could eventually provide an opportunity to assess how the presence of an extra chromosome affects early embryology and, potentially, neural induction (Oldak et al., 2023; Pedroza et al., 2023; Weatherbee et al., 2023). We used isogenic T21 and euploid control iPSCs to avoid natural genetic variation between individuals that generates transcriptomic variation, enabling accurate assignment of causal relationships between genotype and gene expression (Weick et al., 2013; Murray et al., 2015). To our knowledge, this is the first study to explore the earliest stages of neural induction in T21.

Of the 300 proteins encoded on Hsa21, many are dosage sensitive that means their gene dosage may be driving phenotypes in DS. However, we do not have a complete picture of which of these 300 protein-coding genes are or are not dose sensitive, making it difficult to attribute DS phenotypes to any one gene or small groups of genes (e.g., drivers). In our analysis, we were somewhat surprised to find that only a small number of Hsa21 genes have altered expression. Our results may be due to our stringent parameters or differences in stage of development. Other transcriptomic studies focused on Down syndrome and neural/brain development observe larger proportions of Hsa21 genes as DEGs, albeit at later stages (Olmos-Serrano et al., 2016; Sobol et al., 2019; Li et al., 2022; Meharena et al., 2022). However, our results align with previous studies of T21 iPSC derived NPCs and neurons where only 10% of Hsa21 genes were upregulated in NPCs and expression of 20–30% more Hsa21 genes increased at the neuron time point. Together these results indicate that few Hsa21 genes are overexpressed at very early developmental times, and more Hsa21 genes are overexpressed as development progresses.

It is also possible that T21 leads to imbalanced representation of different cell types (e.g., early progenitors vs. more differentiated progenitors) which could explain downregulation of TSPEAR, OLIG2, SIM2 and CLIC6 expression in T21 cells. Single cell analysis is required to assess the cell type differences in our culture. In fact, our previous single cell analysis of T21 interneuron progenitors did indicate an enriched population of a progenitor subtype in T21 (Giffin-Rao et al., 2022) and so it would be interesting to see if there is a similar divergence of cells in T21 in neural induction.

The low percentage of affected Hsa21 genes and early global transcriptomic changes during neural induction suggest mechanisms beyond Hsa21 specific genes as drivers of early neurodevelopment defects. The results also suggest that overexpression of a few “driver” genes on Hsa21 is sufficient to initiate genome wide expression dysregulation. Candidate driver Hsa21 genes that emerge from our analysis include those that are overexpressed in T21 cells at the earliest time point (Figure 3B) as well as TIAM1 that emerges when we use less stringent analysis parameters (Figure 6).

TIAM1 is a mediator of Disheveled and Rac1 interaction required for Wnt5a-induced signaling transduction (Čajánek et al., 2013). Knockdown of TIAM1 causes impaired neuron generation (Čajánek et al., 2013), suggesting that upregulation in T21 may force early neural differentiation compared to euploid cells. The specific contribution of TIAM1 upregulation in T21 on neural differentiation will need to be further explored.

We found only three Hsa21 genes—*CHODL*, *PCP4*, and *TTC3* –were consistently altered throughout the process of neural induction. All three have roles in neural development and function, implicated in processes, such as neurite outgrowth, that are related to neuron differentiation and maturation (Berto et al., 2007; Mouton-Liger et al., 2011; Zhong et al., 2012; Berto et al., 2014; Mouton-Liger et al., 2014; Sleigh et al., 2014; Kitazono et al., 2020; Endo et al., 2023). The increased expression of these genes in T21 cells as they differentiate may suggest that T21 cells are undergoing heterochronic, or early, differentiation as has been suggested by other transcriptomic studies (Li et al., 2022; Zhou et al., 2023).

*TTC3* has also been implicated in pathways crucial for proper maintenance of the proteasome, including the ribosomal quality control (RQC) system (Endo et al., 2023), and the ubiquitin-proteasome system (Zhou et al., 2022), which also appeared in our functional analysis in Cluster 3 (Figure 4C) Overexpression of *TTC3* can aggregate TTC3 protein, resulting in loss of its physiologic ubiquitin-ligase activity. This aggregation may simultaneously promote aggregation of pathological proteins, such as amyloid beta, while also causing inappropriate segregation of proteins crucial for mitochondrial function, such as DNA polymerase γ (POLG) (Zhou et al., 2022). During periods of translational stress when TTC3 is physiologically upregulated, it interacts with the 40S ribosomal subunit to repress translation of transcripts associated with metabolic processes required for cellular homeostasis (Endo et al., 2023). Taken together, this early upregulation of TTC3 may contribute to known metabolic dysfunction through promotion of mitochondrial stress and downregulation of key homeostatic processes and later protein aggregation associated with Alzheimer’s disease development in individuals with T21 (Lanzillotta and Di Domenico, 2021). However, future work is needed to determine if TTC3 is a direct regulator of metabolic stress in T21 and to determine if TTC3 upregulation is an effect of gene dosage or is an early indication of broader trisomy associated translational stress.

### 4.2 Emergence of dysregulated pathways previously implicated in Down syndrome during neural induction

Reductions in cortical size, cellularity and neurogenesis have been established in mid-late gestation fetuses and neonates with T21 (Ross et al., 1984; Wisniewski et al., 1984; Schmidt-Sidor et al., 1990; Golden and Hyman, 1994; Guihard-Costa et al., 2006; Guidi et al., 2008; Guidi et al., 2011; Guidi et al., 2018; Stagni et al., 2019; Baburamani et al., 2020; Patkee et al., 2020; Tarui et al., 2020; McCann et al., 2021). Several non-exclusive hypotheses have emerged to explain these deficits including that T21 results in fewer progenitors and/or reduced progenitor proliferation, decreased migration of progenitors, or increased progenitor death (Lu et al., 2012; Huo et al., 2018; Giffin-Rao et al., 2022). Our results shed some mechanistic light on the impact of T21 on the establishment of the nervous system, and demonstrate that T21 dysregulates genes involved in pathways relevant to the phenotypes of individuals with DS and cells from these individuals including oxidative stress, morphological abnormalities, immune disorders, divergent metabolic states, and premature aging (Coskun and Busciglio, 2012; Muchová et al., 2014).

Divergent metabolic states impact neurogenesis (Zheng et al., 2016; Iwata and Vanderhaeghen, 2021; Traxler et al., 2021). We observe an increased regulation of Positive Cellular Metabolic Process and Positive Regulation of Reactive Oxygen Species Metabolic Process as potential drivers for deficits in neural development in T21. These findings are supported by previous analyses of T21 neural progenitor cells having increase metabolic requirements due to mitochondrial stress (Prutton et al., 2023) and increased oxidative stress (Jovanovic et al., 1998). Importantly, our data indicate that T21 driven metabolic dysfunction emerges early in neural development and thus may affect altered neurogenesis in DS.

Our results suggest that ECM pathways are aberrant in DS. During neural development, neural stem cells and progenitors must precisely regulate their morphology to allow for proper function. The ECM is a key regulator in this process, modulating cell adhesion, shape, proliferation, differentiation, migration and morphogenesis of neural tissues (Long and Huttner, 2019). The Hsa21 gene RUNX1 has been implicated in the regulation of the extracellular matrix in DS leading to a decrease in cell migration (Mollo et al., 2022). T21 driven increased cell-cell adhesion early in neural induction may lead to reduced migration and proliferation, ultimately affecting the process of neural development.

Overall, our results unveil early dysregulation of pathways that have been previously implicated in neural development and function in DS. Increases in oxidative stress and interferons, as well as cell death, likely impact the generation of progenitors and neuron differentiation. Manipulation of these specific cellular processes in early T21 neural progenitors is needed to link metabolism with neurogenesis in T21.

### 4.3 WNT genes are dysregulated by T21

WNTs are a highly conserved group of secreted factors that function in a tightly regulated temporal- and spatial-specific manner during forebrain development to regulate proliferation, differentiation, and regional identity (Clevers, 2006; Mulligan and Cheyette, 2012; Tiberi et al., 2012). WNT signaling controls initial formation of the neural plate and subsequent patterning decisions in the embryonic nervous system (Patapoutian and Reichardt, 2000; Leung et al., 2016). WNTs signal through β-catenin in the canonical pathway, or through non-canonical pathways that mediate calcium (Liu et al., 2022). The distinct contribution of these two modes of WNT signaling on stem cell maintenance and neural differentiation is confounded by the promiscuity of ligands and receptors across pathways and cross talk with other signaling pathways (Bengoa-Vergniory and Kypta, 2015). Our transcriptional data indicate that WNT signaling gene expression is dysregulated in T21 during neural induction (Figures 2, 5, 6).

Our control data are consistent with the existing body of literature showing a decrease in canonical WNT pathway signaling over the course of neural induction (Heeg-Truesdell and LaBonne, 2006; Huang et al., 2016; Telias and Ben-Yosef, 2021). Canonical WNT signaling promotes stem cell proliferation and inhibition of progenitor apoptosis (Dravid et al., 2005; Davidson et al., 2007; Lee et al., 2015). The decrease in canonical WNT signaling in our results correlates with decreased cell proliferation in T21 (Sobol et al., 2019; Sharma et al., 2022). Overall, given the critical role of the canonical WNT signaling pathway in maintaining stemness and proliferation, these data may indicate that T21 cells are less responsive to cues to maintain or expand progenitor pools in the brain. It will be important to test whether WNT activation alters early neural progenitor subpopulations through further study of T21 cell cycle and differentiation dynamics following selective canonical agonism with small molecule inhibitors like CHIR99021 or over expression of non-degradable β-catenin.

These data are consistent with previous work by our lab and others showing a downregulation of canonical WNT signaling in T21 cells (Hibaoui et al., 2014; Granno et al., 2019; Giffin-Rao et al., 2022; Qiu et al., 2023). DYRK1A, a Hsa21 dosage sensitive gene, interacts with the canonical pathway through GSK3β to inhibit pathway activity (Hibaoui et al., 2014; Qiu et al., 2023). However, our data with stringent analysis parameters does not show consistent upregulation of DYRK1A in trisomic cells during neural induction suggesting that T21 may have a broader effect on the canonical WNT pathway beyond DYRK1A.

## 5 Conclusion

This comprehensive analysis reveals that trisomy 21 impacts discrete developmental pathways at the earliest stages of neural development. Further, the results suggest that metabolic dysfunction arises early in embryogenesis in trisomy 21 and may thus affect development and function more broadly.

## Data availability statement

The data presented in the study are deposited in the GEO repository, accession number GSE247990.

## Ethics statement

The studies involving cells from humans were approved by the University of Wisconsin-Madison Institutional Review Board. The studies were conducted in accordance with the local legislation and institutional requirements. The human samples used in this study were acquired from as part of a previous study for which ethical approval was obtained.

## Author contributions

JM: Conceptualization, Writing – original draft, Writing – review and editing, Data curation, Formal Analysis, Investigation, Methodology, Software, Visualization. JP: Data curation, Formal Analysis, Investigation, Methodology, Visualization, Writing – original draft, Writing – review and editing. MC: Data curation, Formal Analysis, Investigation, Methodology, Writing – review and editing. IS: Data curation, Formal Analysis, Methodology, Writing – review and editing. NM: Data curation, Methodology, Writing – review and editing. CS: Methodology, Writing – review and editing. YG-R: Methodology, Writing – review and editing, Data curation, Formal Analysis. AB: Data curation, Formal Analysis, Writing – review and editing, Conceptualization, Funding acquisition, Project administration, Supervision, Writing – original draft.

## Footnotes

1
https://www.r-project.org

2
http://amp.pharm.mssm.edu/Enrichr

## Supplementary material

Supplementary Figure 1 Gene expression differences between isogenic trisomy 21 and euploid cells at each time point. Principal Component Analysis of trisomy 21 (red) and isogenic control (black), showing PC1 variance as condition at **(A)** Day 6, **(B)** day 10, and **(C)** day 17. Heatmaps of expression profiles of T21 vs. isogenic control at each timepoint. Time course replicates are labeled with light gray for day 6, medium gray day 10, and dark gray day 17. Isogenic control is represented by black and T21 by red. Upregulated genes are displayed in red while downregulated genes are expressed in blue.
