# Supplementary figures and images for: Transcriptional consequences of trisomy 21 on neural induction
Source: Front Cell Neurosci. 2024 Jan 30;18:1341141. doi: 10.3389/fncel.2024.1341141 (PMC10865501; doi:10.3389/fncel.2024.1341141)

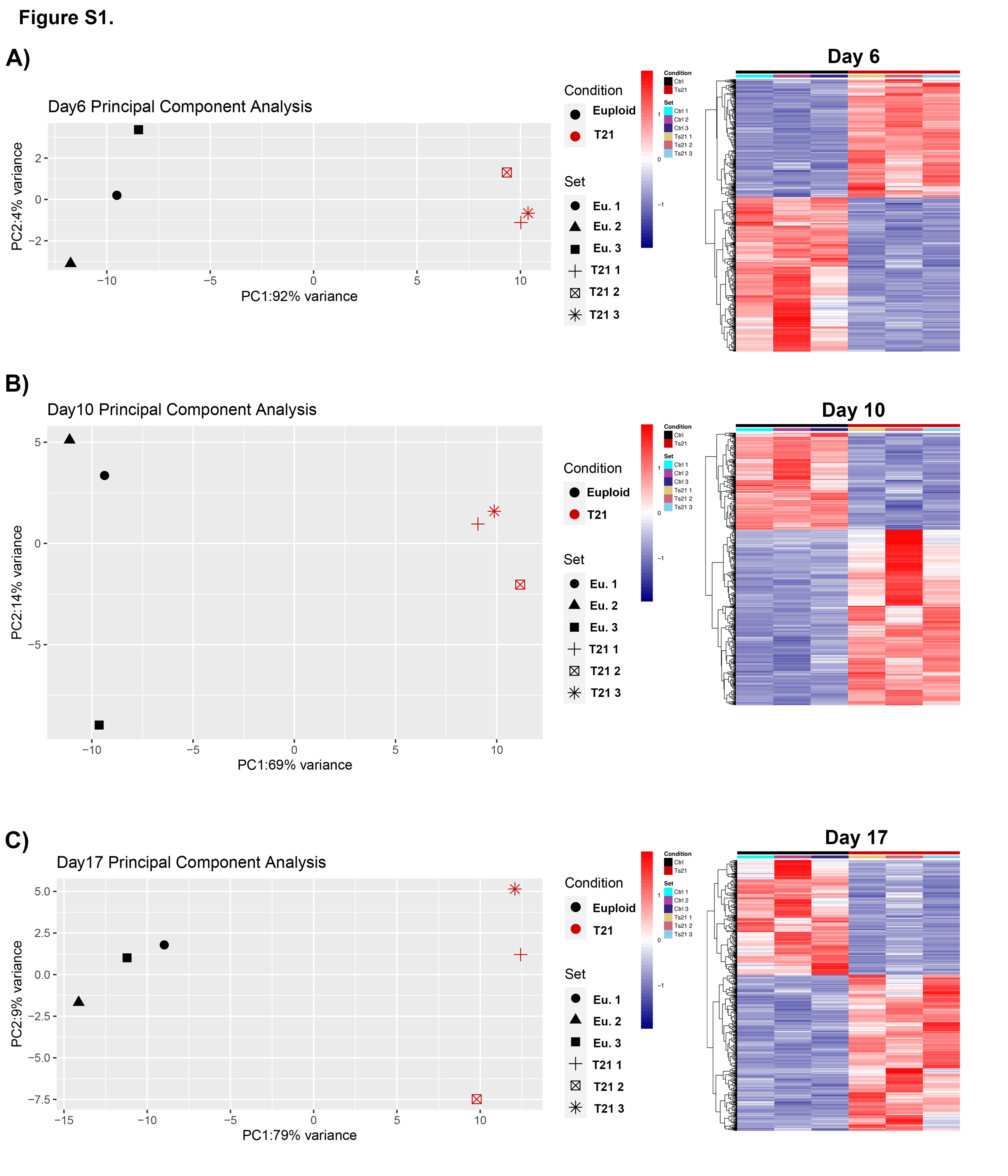

Supplement: Supplementary Figure 1 — Gene expression differences between isogenic trisomy 21 and euploid cells at each time point. Principal Component Analysis of trisomy 21 (red) and isogenic control (black), showing PC1 variance as condition at (A) Day 6, (B) day 10, and (C) day 17. Heatmaps of expression profiles of T21 vs. isogenic control at each timepoint. Time course replicates are labeled with light gray for day 6, medium gray day 10, and dark gray day 17. Isogenic control is represented by black and T21 by red. Upregulated genes are displayed in red while downregulated genes are expressed in blue. [file Image_1.JPEG]
